# Increasing follow-up questionnaire response rates in a randomized controlled trial of telehealth for depression: three embedded controlled studies

**DOI:** 10.1186/s13063-016-1234-3

**Published:** 2016-02-24

**Authors:** Louisa Edwards, Chris Salisbury, Kimberley Horspool, Alexis Foster, Katy Garner, Alan A. Montgomery

**Affiliations:** Centre for Academic Primary Care, School of Social and Community Medicine, University of Bristol, Canynge Hall, 39 Whatley Road, Bristol, BS8 2PS UK; School of Health and Related Research (ScHARR), University of Sheffield, Regent Court, 30 Regent Street, Sheffield, S1 4DA UK; Nottingham Clinical Trials Unit, The University of Nottingham, C Floor, South Block, Queen’s Medical Centre, Nottingham, NG7 2UH UK

**Keywords:** Depression, Email reminders, Embedded study, Photographs, Pre-notification, Recruitment, Response rates, Retention, Telehealth, Trials

## Abstract

**Background:**

Attrition is problematic in trials, and may be exacerbated in longer studies, telehealth trials and participants with depression – three features of The Healthlines Study. Advance notification, including a photograph and using action-oriented email subject lines might increase response rates, but require further investigation. We examined the effectiveness of these interventions in three embedded Healthlines studies.

**Methods:**

Based in different trial sites, participants with depression were alternately allocated to be pre-called or not ahead of the 8-month follow-up questionnaire (Study 1), randomized to receive a research team photograph or not with their 12-month questionnaire (Study 2), and randomized to receive an action-oriented (‘ACTION REQUIRED’) or standard (‘Questionnaire reminder’) 12-month email reminder (Study 3). Participants could complete online or postal questionnaires, and received up to five questionnaire reminders. The primary outcome was completion of the Patient Health Questionnaire (PHQ-9). Secondary outcome measures were the number of reminders and time to questionnaire completion.

**Results:**

Of a total of 609 Healthlines depression participants, 190, 251 and 231 participants were included in Studies 1–3 (intervention: 95, 126 and 115), respectively. Outcome completion was ≥90 % across studies, with no differences between trial arms (Study 1: OR 0.38, 95 % CI 0.07–2.10; Study 2: OR 0.84, 95 % CI 0.26–2.66; Study 3: OR 0.53 95 % CI 0.19–1.49). Pre-called participants were less likely to require a reminder (48.4 % vs 62.1 %, OR 0.41, 95 % CI 0.21–0.78), required fewer reminders (adjusted difference in means −0.67, 95 % CI −1.13 to −0.20), and completed follow-up quicker (median 8 vs 15 days, HR 1.35, 95 % CI 1.00–1.82) than control subjects. There were no significant between-group differences in Studies 2 or 3.

**Conclusions:**

Eventual response rates in this trial were high, with no further improvement from these interventions. While the photograph and email interventions were ineffective, pre-calling participants reduced time to completion. This strategy might be helpful when the timing of study completion is important. Researchers perceived a substantial benefit from the reduction in reminders with pre-calling, despite no overall decrease in net effort after accounting for pre-notification.

**Trial registration:**

Current Clinical Trials ISRCTN14172341

**Electronic supplementary material:**

The online version of this article (doi:10.1186/s13063-016-1234-3) contains supplementary material, which is available to authorized users.

## Background

The ability to detect treatment effects in a randomized controlled trial is partly determined by the number of participants providing primary outcome data, which is often threatened by participant attrition. Therefore, trial protocols specify the expected loss to follow-up and the number of participants required for recruitment in order to compensate for this attrition. However, primary outcome data are often collected in trials through postal or online questionnaires, whereby meeting target response rates can be difficult. One explanation is that questionnaires might be overlooked or ignored by participants, owing to the volume of junk mail via post and email.

One of the largest pragmatic telehealth trials to date, The Healthlines Study, involved two linked, parallel, randomized controlled trials which sought to assess the effectiveness and cost-effectiveness of a telehealth intervention to support patients with two exemplar and common long-term conditions: depression (*n* = 609) and increased risk of cardiovascular disease (*n* = 641) [1]. The primary outcome in the depression trial was positive response to treatment, as measured by improvement on the Patient Health Questionnaire (PHQ-9) [2] at four months post-randomization, with additional follow-up questionnaires administered at eight and 12 months. Participants could choose to complete questionnaires online or by post, and received up to five questionnaire reminders. Approximately 3–4 months into the start of the 8-month follow-up in the depression trial, it was apparent that the response rate (approximately 60–70 %) was not only falling below the 4-month rate for the equivalent time period (approximately 85 %), but below the trial protocol target of 80 %. This downward trend was not evident in the cardiovascular disease trial, and so three simple interventions were introduced to try to improve response rates amongst the depression group alone.

Achieving high response rates might be particularly difficult in trials with longer follow-up periods [3, 4] and telehealth trials [5], as well as with patients with depression [6, 7], who typically struggle with concentration and motivation. In these instances, the efficiency gained by simply sending out follow-up questionnaires when they are due might not outweigh the effort required from multiple, time-consuming completion reminders or eventual loss to follow-up. Instead, it might be more effective to employ strategies when sending out questionnaires, to increase response rates. Therefore, we investigated different strategies embedded within the depression trial of The Healthlines Study to boost response rates.

Given the risk of bias from follow-up attrition and, hence, the threat to validity and reliability [8], it is not surprising that numerous strategies for increasing response rates to follow-up questionnaires have been developed and tested. Some of these strategies are resource-intensive, but generally effective [9–12]. One popular resource-intensive strategy is contacting participants to give them advance notice about an upcoming questionnaire (i.e., pre-notification). In two systematic reviews, which included 28 [10] and 47 trials [9] employing this strategy, Edwards et al. demonstrated that pre-notification increased the odds of responding by about half. However, many of the studies in these reviews were nested within a variety of study designs. Furthermore, the reviews included studies in both healthcare and non-healthcare settings, and examined this intervention in relation to completing postal questionnaires alone [9, 10]. Further investigation is required regarding whether the beneficial pre-notification effect will transfer to a trial of mixed completion methods, in which participants can choose to complete either postal or online questionnaires. Therefore, we evaluated response rate effects in those completing either postal or online questionnaires after eight months of participation in The Healthlines Study depression trial.

Another broad strategy that has been examined in several studies seeks to capture the attention of participants by adding some form of novelty or distinctiveness to the questionnaire or accompanying cover letter [9, 10]. The reduction in researcher time and effort to carry out such response rate strategies is an obvious benefit over more resource-intensive methods, but the success in achieving heightened response rates is less clear. One Cochrane review noted that including a picture within the questionnaire cover letter tripled the odds of response amongst participants completing online surveys (two trials), whereas this same intervention seemed to have no reliable effect for those receiving a postal copy (four trials) [9]. The differential effect might be because the emailed photographs were in colour and contained actual people [13], which could have enhanced their distinctiveness and visual appeal [14], whereas the pictures within the cover letter of the postal questionnaires appeared to be in black and white [15] and included a mixture of photographs and drawings or graphics [15–18]. Additionally, none of these studies recruited participants from primary care or were embedded within a host trial. Thus, we investigated whether a colour photograph containing the names of the local research team, with whom all participants had had some degree of contact, in the cover letter or email prompting questionnaire completion would boost 12-month follow-up questionnaire responses in the depression trial of The Healthlines Study.

A relatively easy and cost-effective means of reminding non-responders to complete questionnaires is through email reminders. Some research has examined altering email subject lines [9], since this might affect whether an email is opened at all [19, 20]. Although it only involved two trial comparisons, one Cochrane review found no evidence of an effect on the odds of responding when comparing a topic in the subject email line against a blank subject line, including ‘Survey’ in the subject line against a blank subject line, or even including a plea for help in the subject line or not [9]. Since these comparisons involved a student sample, the findings might not extrapolate to participants in a health-related trial. We trialled whether an action-oriented email reminder subject line, containing visual distinctiveness with some words capitalized (‘ACTION REQUIRED’), might boost 12-month response rates in non-responders in the depression trial when compared with the reminder subject line (‘Questionnaire reminder’) used at previous follow-up time points within The Healthlines Study.

Taken together, the three embedded response rate studies – pre-calling, including a team photo, and using an action-oriented email reminder – attempt to address some of the shortcomings we noted in the previous studies, but do so in a host trial that encompasses study characteristics particularly subject to participant attrition – longer follow-up (12 months), a telehealth-based design, and in participants experiencing depression. We hypothesized that receiving each of these interventions would improve response rates, as well as reduce questionnaire reminders and response time to the questionnaire over those who received the standard study procedure.

## Methods

### Overview of The Healthlines Study

The Healthlines Study comprised two linked, parallel randomized controlled trials of patients with depression and raised risk of cardiovascular disease who were allocated to a telehealth intervention plus usual care or usual care alone [1]. The multicentre trials were conducted in and around Bristol, Sheffield and Southampton, UK. Delivered by NHS Direct Health Information Advisors, the intervention included telephone-based support and advice, the use of online tools and resources (e.g., computerized cognitive behavioural therapy), and motivational interviewing. In the depression trial, intervention participants received regular telephone coaching approximately every two weeks for a maximum of eight telephone sessions. The majority of telephone contact occurred in the first four months, but participants could remain in the trial for up to 12 months. The trials were approved by the National Research Ethics Service Committee South West – Frenchay (Reference 12/SW/0009), participants gave written informed consent, and the depression trial is registered with Current Controlled Trials (ISRCTN14172341; registered, 26 June 2012). Since the current interventions involve the depression group alone, we focus solely on this trial in this paper.

### Recruitment to the depression trial of The Healthlines Study

Using an anonymized, code-based record search, participants were recruited to the Healthlines depression trial from 43 general practices between June 2012 and July 2013. Basic inclusion criteria were age ≥18 years, telephone and internet access, researcher-confirmed diagnosis of depression using the Clinical Interview Schedule – Revised [21], and PHQ-9 score ≥10 [2]. A number of exclusion criteria were applied, such as having a severe cognitive impairment, already receiving formal psychological therapy and substance dependency. The study invitation pack was posted by GP practice staff, with expressions of interest returned to the research team directly. After eligibility was confirmed over the telephone by researchers, interested participants completed a consent form and baseline questionnaire either online or via a postal copy, as they preferred. In total, 609 participants (307 intervention, 302 usual care) were randomized to the depression trial, of which 318, 233 and 58 were from the areas around Bristol, Sheffield and Southampton, respectively.

### Standard follow-up procedures in the host trial

Participants in the depression trial were followed up with a questionnaire at three time points: 4, 8, and 12 months after randomization. All follow-up questionnaires included the same questions (e.g., depression (PHQ-9), anxiety, treatment satisfaction, health service use), and took approximately 40 minutes to complete. A few days before the 4-month follow-up was due, participants were automatically sent the questionnaire in the same format (online or postal) that they had chosen to complete at baseline.

**Fig. 1:**
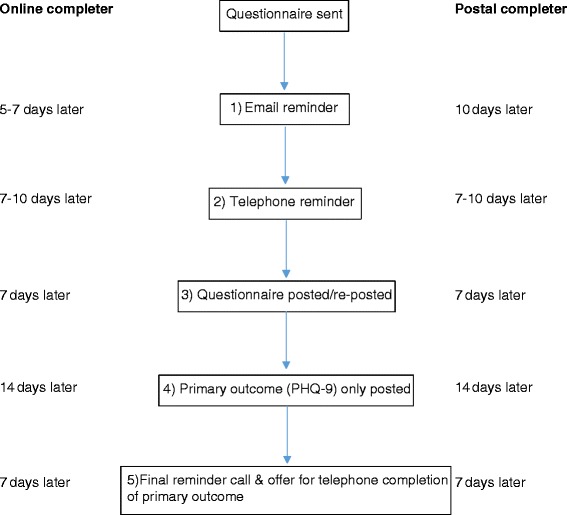
Schematic of follow-up questionnaire reminder steps and timings for online and postal completers

For those who did not respond promptly, a standard procedure was followed in terms of sending out reminders for all follow-ups (see Fig. 1). This involved the following sequence of steps: sending an email reminder; phoning the participant; posting a questionnaire (a second copy was posted for those who were completing the paper version); posting just the primary outcome questions (the PHQ-9); finally, phoning participants to ask them to post back the PHQ-9 or offering to complete it over the phone. After this final phone reminder was completed, no further attempts were made to collect data for that follow-up time point. Note that in order to maximize the likelihood that the reminder email would be opened by the participant, the reminder email subject line (‘The Healthlines Study – Questionnaire reminder’) included both the study name and the purpose of the email. This helped to ensure that the reminder message was received by participants, even if they failed to open the email itself. Although the order of reminders was the same for all participants and follow-ups, the timing of the reminders varied slightly by completion method (see Fig. 1).

### Overview of the embedded interventions

The following three studies are individually embedded within the Healthlines depression host trial, and separately compare an introduced response rate intervention to the standard follow-up questionnaire protocol method just described. Each of these studies seeks to investigate the impact on follow-up questionnaire response rates through pre-calling participants or not (Study 1), personalizing study materials by including a colour photograph of the research team within the questionnaire cover letter or email versus not including a photo (Study 2), and using an urgent action subject line (‘ACTION REQUIRED’) in the initial email reminder versus one that simply states it is a reminder (‘Questionnaire reminder’) (Study 3) (see Additional file 1).

Study 1 occurred at the 8-month follow-up time point. Based on an early and informal indication that there was a benefit from pre-calling participants (this group seemed to require fewer reminders), this procedure was adopted uniformly for all final, 12-month follow-ups. However, because participants were recruited over a period of several months, some participants were sent 12-month follow-up questionnaires before others had received their 8-month follow-up questionnaires, and so this decision was made before any analyses had been performed. Therefore, Studies 2 and 3 include the pre-call intervention from Study 1 as an adopted standard procedure, but examine the possibility of further boosting the 12-month follow-up response rate with two separate interventions in different study centres (see Additional file 2 for CONSORT checklist).

#### Study 1: advance notification through pre-calling study

The pre-calling intervention was devised in response to the lower than expected 8-month follow-up questionnaire completion rate in the first recruiting study centre (Bristol), and so was introduced six months after the first 8-month follow-up questionnaire was sent out. In ascending randomization date order, the remaining participants requiring the 8-month follow-up between September 2013 and March 2014 were alternately allocated to one of theo groups; they either received an advance notification telephone call (i.e., a pre-call) from a researcher one to three days ahead of being sent the questionnaire (intervention group) or they were simply emailed or posted the questionnaire without a telephone call (control group). The decision to email or post the questionnaire to control participants was based on how they had completed the questionnaire previously. During the intervention pre-call, participants were informed that the next study questionnaire was due, were asked whether they would like to complete it online or via post, and were encouraged to return the questionnaire within 10 days, and their contact details were verified (see Additional file 1).

Participants were often difficult to contact by telephone. Multiple attempts were made to contact intervention participants at different times of day over the course of the days leading up to when the 8-month follow-up was due. If direct telephone contact could not be made ahead of this due date, the questionnaire was sent to the participant based on their previous completion method (online or postal). In these instances, the research team left a voicemail message (or emailed or texted the same information, if voicemail was not possible) which communicated the same information that would have been relayed had direct communication been achieved, along with the researcher’s contact details.

All other procedures were identical for intervention and control participants. Questionnaire reminders, when required, followed the standard procedure outlined earlier (see Fig. 1).

#### Study 2: research team photo study

Of the participants with depression from the Bristol study centre who were due to complete the 12-month follow-up questionnaire between October 2013 and July 2014, half were randomized to receive a cover letter with a colour photo of the Bristol research team (intervention group) and half were randomized to receive the standard, black and white cover letter without a photo (control group) (see Additional file 1). In this embedded parallel group randomized controlled trial, the local researcher used simple randomization (1:1) by computing a random number in Excel for each participant, sorting the data by random number, and then allocating the first half of participants to the intervention group and the remaining half to the control group.

Participants approaching the 12-month follow-up received an advance notification telephone call from the local researcher one to three days ahead of the due date for this questionnaire. The phone conversation was similar to Study 1, except that participants were informed that they would receive an email thank you note with more details about the study upon completing the final questionnaire. If the researcher was unable to contact the participant, the same procedure as in Study 1 was followed.

We reasoned that a photo by itself was probably not very informative, but that if participants were able to link the name of the particular research team member or members that they had spoken to over the course of the study to the picture, this would increase the effect of the photo. Therefore, underneath the photo, the names of each of the five Bristol research team members were listed. To ensure maximum visibility, the photo was located in the upper right corner of the cover letter that was posted to each participant with either their questionnaire (paper-based) or with instructions around completing the online questionnaire. For participants who had opted to complete the online questionnaire, the same photo was also included at the end of the email that was sent with instructions on accessing the online survey (some email providers might automatically include the photo as an attachment, however). Since we wanted to ensure that the survey weblink and access details were not missed and the email was not deleted on seeing a photo of people the participant would not recognise, we placed the photo at the end of the email, close to the survey weblink. Finally, the same photo was incorporated into each reminder letter or email sent to intervention participants (Fig. 1).

Participants in the control group received the same study information as the intervention group, but without a photo. This was the standard cover letter or email that had been sent to all participants at the previous follow-up time points. As required, the reminder procedure as outlined in Fig. 1 was followed.

#### Study 3: action-oriented email reminder subject line study

In this embedded parallel group randomized controlled trial, approximately half of the participants with depression from the Sheffield and Southampton study sites who were due to complete the 12-month follow-up questionnaire between November 2013 and July 2014 were randomized 1:1 to receive either the intervention reminder email subject line (‘The Healthlines Study – ACTION REQUIRED’) or the standard reminder email subject line (‘The Healthlines Study – Questionnaire reminder’) (see Additional file 1). Study 3 used the same randomization procedure as Study 2.

As before, participants first received the advance notification call as described in Study 2, and were sent the questionnaire via their preferred completion method (online or postal). In line with the usual reminder protocol, if the completed questionnaire had not been returned within 5–7 days for online completers or 10 days for postal completers, participants were sent a reminder email from the local researcher using the randomly allocated subject line. Other than the email subject line intervention, the content of the reminder email was identical for both groups and the same as for previous follow-ups.

Since some participants at earlier follow-up time points said that they had overlooked the email in their inbox, the manipulated subject line included the new, action-oriented words in capitals, in order to increase the visual salience of the email. We expected that the use of directive, action-oriented language would increase the participant’s perception that reading the email and returning the completed questionnaire was of greater importance than the standard email reminders that they might have received with previous follow-ups. Given that the email subject line had changed for these participants, its novelty and distinctiveness should also be attention grabbing.

### Sample size

Since these were embedded studies, in which there were practical constraints around the timing of implementing the interventions in different trial centres, we did not derive a formal sample size calculation *a priori*. Instead, sample size was determined by these constraints.

### Primary and secondary outcomes

The primary outcome for the embedded studies was completion of the primary outcome (PHQ-9) questionnaire within 3 months. Secondary outcome measures included the total number of questionnaire reminders required (range: 0–5), as well as the time (in days) taken to complete and return the questionnaire, calculated as the difference between the date it was sent and date it was received.

### Statistical analysis

**Fig. 2:**
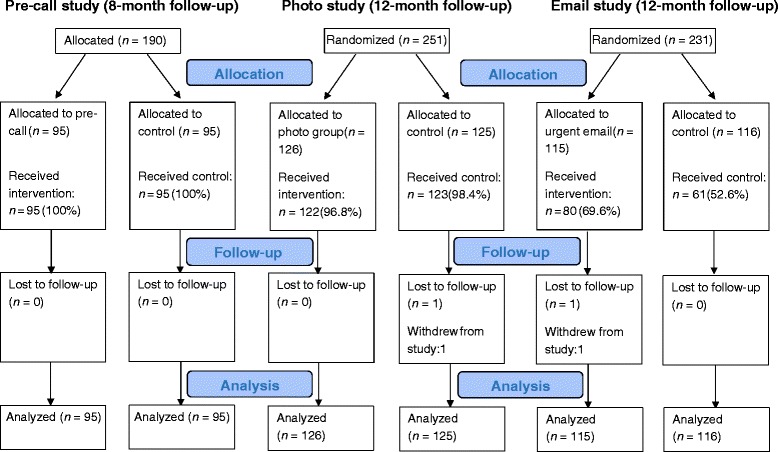
CONSORT flow diagram for the pre-call, photo and email intervention studies

First, appropriate descriptive statistics (mean, standard deviation (SD), frequencies) were used to summarize the baseline characteristics of participants. In each of the embedded studies, the primary intention-to-treat analysis investigated whether the particular response rate intervention had an effect on questionnaire completion. This was examined with unadjusted and adjusted logistic regression models (with participant sex, baseline age and PHQ-9 score, main Healthlines Study trial allocation arm and questionnaire method sent (paper or online) as covariates in the adjusted models). Since not all randomized participants received the response rate intervention in the photo and email reminder studies (see Fig. 2), either because they opted to complete the PHQ-9 questionnaire over the phone during the pre-call or because they responded before the first questionnaire reminder was due, sensitivity analyses were carried out on the primary outcome after excluding these participants. Next, secondary outcomes were analyzed using linear (number of questionnaire reminders received) and Cox regressions (days to questionnaire completion). Again, both unadjusted and adjusted models were tested, and sensitivity analyses were conducted where appropriate. Analyses were carried out using Stata13.1 (StataCorp).

## Results

### Study 1: pre-call study

To mitigate response rate concerns, the pre-call intervention was devised and introduced partway through the 8-month follow-up. After excluding those participants who had already had their 8-month follow-up by this point (*n* = 114) or had withdrawn from the main host trial (*n* = 14), 190 of the 318 Healthlines Study depression participants from the Bristol study centre were alternately allocated to the pre-call study between September 2013 and March 2014. Of these, 95 participants were allocated to receive a pre-call ahead of being sent the 8-month follow-up questionnaire (intervention group) and 95 were sent the questionnaire without a telephone call (control group) (see Fig. 2). As shown in Table 1, the two groups were well balanced in all of the participant characteristics.

**Table 1:** Participant characteristics by allocated response rate intervention group in the three embedded studies

	8-month follow-up^a^ (pre-call)		12-month follow-up^a^ (photo)		12-month follow-up^b^ (email)	
	Control (*n* = 95)	Pre-called (*n* = 95)	Control (*n* = 125)	Photo (*n* = 126)	Control (*n* = 116)	Urgent email (*n* = 115)
Baseline age, mean (standard deviation)	50.5 (13.1)	49.7 (13.9)	49.2 (12.6)	50.0 (14.0)	48.6 (11.4)	50.0 (12.3)
Female	68 (72)	66 (69)	89 (71)	86 (68)	73 (63)	77 (67)
Baseline Patient Health Questionnaire (PHQ-9) score	16.4 (5.0)	17.0 (4.5)	17.2 (4.9)	16.7 (4.6)	16.9 (4.8)	16.7 (4.5)
Main trial arm						
Intervention	42 (44)	51 (54)	64 (51)	59 (47)	51 (44)	61 (53)
Questionnaire completion method						
Online	36 (38)	38 (40)	57 (46)	54 (43)	46 (40)	33 (29)
Paper	42 (44)	44 (46)	54 (43)	50 (40)	47 (41)	59 (51)
Patient Health Questionnaire (PHQ-9) only (paper or phone)	15 (16)	8 (8)	7 (6)	14 (11)	16 (14)	12 (10)
Not returned or withdrawn	2 (2)	5 (5)	7 (6)	8 (6)	7 (6)	11 (10)

Data are reported as number (percentage), unless otherwise indicated

^a^Based on participants recruited from the Bristol area only

^b^Based on participants recruited from the Sheffield and Southampton areas

**Fig. 3:**
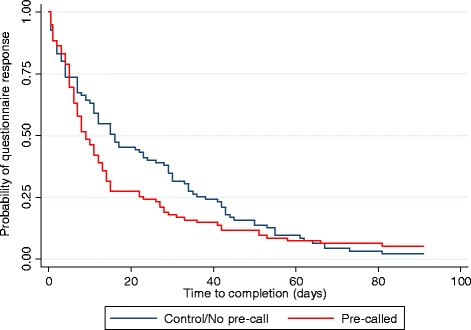
Kaplan–Meier curve of time to questionnaire completion by pre-call intervention group

As noted in the introduction, despite early indications that questionnaire completion was falling below the 4-month follow-up questionnaire levels, the final 8-month completion rate for the pre-call study was extremely high in both arms (95 % or greater; see Table 2). Given this ceiling effect, it is not surprising that there was no evidence of a benefit of pre-calling participants in terms of returned questionnaires in the primary analyses (see Table 2). However, participants in the pre-calling arm were less likely to require a reminder (48.4 % vs 62.1 %, odds ratio (OR) 0.41, 95 % confidence interval (CI) 0.21–0.78), required fewer reminders (adjusted difference in means −0.67, 95 % CI −1.13 to −0.20), and completed follow-up more quickly (median 8 vs 15 days, hazard ratio (HR) 1.35, 95 % CI 1.00–1.82) than participants who received no pre-calling. On average, those in the control group took 22.4 days (SD 20.7) to complete the 8-month follow-up, while those in the pre-called group took just 14.4 days (SD 16.0). These group differences in the number of reminders and length of time to complete the questionnaire can be clearly seen in Fig. 3.

**Table 2:** Effect of pre-call intervention on questionnaire completion, number of questionnaire reminders and questionnaire completion rate

	Control (*n* = 95)	Pre-called (*n* = 95)	Unadjusted odds ratio, *b* or hazard ratio	95 % confidence interval; *P*	Adjusted odds ratio, *b* or hazard ratio^a^	95 % confidence interval; *P*
Primary analysis^b^						
Completed questionnaire	93 (98)	90 (95)	0.39	0.07 to 2.05; 0.26	0.38	0.07 to 2.10; 0.27
Secondary analysis^c^						
Number of reminders = 0	36 (37)	49 (52)				
Number of reminders = 1	12 (13)	19 (20)				
Number of reminders = 2	17 (18)	11 (12)				
Number of reminders = 3	13 (14)	4 (4)				
Number of reminders = 4	8 (8)	4 (4)				
Number of reminders = 5	9 (9)	8 (8)	−0.56	−1.03 to −0.09; 0.02	−0.67	−1.13 to −0.20; 0.005
Secondary analysis^d^						
Time to complete questionnaire, median days	15.0	8.0	1.23	0.92 to 1.65; 0.16	1.35	1.00 to 1.82; 0.048

Data are reported as number (percent), unless otherwise indicated. All primary analyses are intention-to-treat analyses

^a^Analyses adjusted by participant sex, baseline age and Patient Health Questionnaire (PHQ-9) score, main trial allocation arm, questionnaire method sent (paper or online)

^b^Logistic regression analysis of pre-call group allocation on whether the 8-month questionnaire was completed or not (odds ratio)

^c^Linear regression of pre-call group allocation on total number of reminders (linear regression coefficient, *b*)

^d^Cox regression of pre-call group allocation on time to complete the questionnaire in days since the questionnaire was sent to the participant (hazard ratio). Zero values are not permitted in the time-to-event analysis, and so 0.5 days were allocated for participants who completed the questionnaire the day it was sent to them, while 91 days were allocated to the seven participants who did not complete the questionnaire. For median: control *n* = 93; pre-called *n* = 90

### Study 2: Team photo study

Of the 251 Healthlines participants with depression from the Bristol study centre who were due to complete the 12-month follow-up questionnaire between October 2013 and July 2014, 126 were randomized to the intervention group (received a cover letter with a colour photo of the Bristol research team) and 125 were randomized to the control group (received the standard cover letter without a photo). As illustrated in Fig. 2, four (3.2 %) intervention and two (1.6 %) control participants did not receive their group allocation, since these participants opted to complete the PHQ-9 alone during the pre-call and before the full questionnaire was sent to them. Once again, the two groups were well balanced in terms of the characteristics shown in Table 1.

Similar to the findings at 8 months, questionnaire completion was impressive, at about 94 % in both arms (see Table 3). There was no evidence that personalizing study materials by including a research team photo resulted in the return of more questionnaires (see Table 3). Unlike the pre-call study findings, similar proportions of those in the intervention and control groups required a reminder (intention-to-treat analysis: 49.2 % vs 45.6 %, OR 1.28, 95 % CI 0.76 to 2.15), and required about the same number of reminders (adjusted difference in means 0.22, 95 % CI −0.13 to 0.56). Participants allocated to receive a team photo (mean 13.8 days (SD 15.9)) took about 1 day longer to complete the questionnaire than the control group (mean 12.9 days (SD 14.9)), although the difference was not significant (see Table 3). As shown in Table 3, there were no notable differences in any of the above findings between the intention-to-treat and sensitivity analyses.

**Table 3:** Effect of photo intervention on questionnaire completion, number of questionnaire reminders, and questionnaire completion rate

	Control	Photo group	Unadjusted odds ratio, *b*, hazard ratio	95 % confidence interval; *P*	Adjusted odds ratio, *b*, hazard ratio^a^	95 % confidence interval; *P*
Primary analysis^b^						
Completed questionnaire	118/125 (94)	118/126 (94)	0.88	0.31 to 2.49; 0.80	0.84	0.26 to 2.66; 0.76
Sensitivity analysis						
Only those actually sent the questionnaire: Number of reminders = 0	116/123 (94)	114/122 (93)	0.86	0.30 to 2.45; 0.78	0.84	0.26 to 2.65; 0.76
Secondary analysis^c^						
Number of reminders = 0	68/125 (54)	64/126 (51)				
Number of reminders = 1	25/125 (20)	27/126 (21)				
Number of reminders = 2	15/125 (12)	15/126 (12)				
Number of reminders = 3	9/125 (7)	6/126 (5)				
Number of reminders = 4	4/125 (3)	5/126 (4)				
Number of reminders = 5	4/125 (3)	9/126 (7)	0.17	−0.19 to 0.52; 0.36	0.22	−0.13 to 0.56; 0.22
Sensitivity analysis^c^						
Only those actually sent the questionnaire: number of reminders = 0	66/123 (54)	60/122 (49)				
Number of reminders = 1	25/123 (20)	27/122 (22)				
Number of reminders = 2	15/123 (12)	15/122 (12)				
Number of reminders = 3	9/123 (7)	6/122 (5)				
Number of reminders = 4	4/123 (3)	5/122 (4)				
Number of reminders = 5	4/123 (3)	9/122 (7)	0.19	−0.17 to 0.55; 0.31	0.23	−0.12 to 0.57; 0.20
Secondary analysis^d^						
Time to complete questionnaire, median days	7.0	9.0	0.93	0.72 to 1.20; 0.57	0.85	0.66 to 1.11; 0.25
Sensitivity analysis^d^						
Only those actually sent the questionnaire: time to complete questionnaire, median days	7.5	10.0	0.91	0.70 to 1.18; 0.49	0.86	0.66 to 1.12; 0.27

Data are reported as number/total (percent), unless otherwise indicated. All primary analyses are intention-to-treat analyses. A sensitivity analysis was carried out on only those participants who were actually sent the questionnaire, as opposed to those who opted to complete the Patient Health Questionnaire (PHQ-9) over the telephone from the outset

^a^Analyses adjusted by participant sex, baseline age and Patient Health Questionnaire (PHQ-9) score, main trial allocation arm, questionnaire method sent (paper or online)

^b^Logistic regression analysis of pre-call group allocation on whether the 8-month questionnaire was completed or not (odds ratio)

^c^Linear regression of pre-call group allocation on total number of reminders (linear regression coefficient, *b*).

^d^Cox regression of pre-call group allocation on time to complete the questionnaire in days since the questionnaire was sent to the participant (hazard ratio). Zero values are not permitted in the time-to-event analysis, and so 0.5 days were allocated for participants who completed the questionnaire the day it was sent to them, while 91 days were allocated to the 15 participants who did not complete the questionnaire. For median: control *n* = 118 (sensitivity *n* = 116); photo *n* = 118 (sensitivity *n* = 114)

### Study 3: Email reminder subject line study

Between November 2013 and July 2014, 231 Healthlines Study participants with depression from the Sheffield and Southampton sites were randomized to receive an action-oriented email reminder subject line (intervention group, *n* = 115) or the standard subject line (control group, *n* = 116). The two groups were fairly well balanced in terms of baseline characteristics and 12-month questionnaire completion method (see Table 1). Thirty-five (30.4 %) intervention and 55 (47.4 %) control participants did not receive their group allocation (see Fig. 2), mainly because these participants completed the questionnaire before a reminder was due (33/35, 94.3 % intervention participants; 52/55, 94.5 % control participants). A further three (2.6 %) control group participants wanted to complete the PHQ-9 questionnaire during the pre-call and before any questionnaire was sent to them, and two (1.7 %) intervention participants received the standard email subject line in error.

Similar to the previous studies, questionnaire completion remained high in this Sheffield and Southampton-based sample, with both trial arms achieving ≥90 % completion (see Table 4). There was no evidence that the email reminder subject line had a benefit on the number of completed questionnaires (see Table 4). Of those participants requiring a reminder, both the intervention and control participants required about the same number of reminders (adjusted difference in means −0.15, 95 % CI −0.65 to 0.36). For those who required a reminder, these participants (sensitivity mean 23.2 days (SD 17.7)) returned the questionnaire about 5 days sooner than the control group (sensitivity mean 28.1 days (SD 17.3)). However, caution is needed in interpreting the time to completion effect, since the confidence interval includes the possibility of a null effect (see Table 4). As in Study 2, there were no notable differences in the pattern of findings between the intention-to-treat and sensitivity analyses.

**Table 4:** Effect of email intervention on questionnaire completion, number of questionnaire reminders, and questionnaire completion rate

	Control	Urgent email	Unadjusted odds ratio, *b* or hazard ratio	95 % confidence interval; *P*	Adjusted odds ratio, *b* or hazard ratio^a^	95 % confidence interval; *P*
Primary analysis^b^						
Completed questionnaire	109/116 (94)	104/115 (90)	0.61	0.23, 1.63; 0.32	0.53	0.19, 1.49; 0.23
Sensitivity analysis						
Only those who required a reminder: Number of reminders = 0	54/61 (89)	69/80 (86)	0.81	0.30, 2.24; 0.69	0.66	0.22, 1.95; 0.45
Secondary analysis^c^						
Number of reminders = 0	55/116 (47)	35/115 (30)				
Number of reminders = 1	26/116 (22)	35/115 (30)				
Number of reminders = 2	10/116 (9)	16/115 (14)				
Number of reminders = 3	7/116 (6)	12/115 (10)				
Number of reminders = 4	7/116 (6)	8/115 (7)				
Number of reminders = 5	11/116 (9)	9/115 (8)	0.27	−0.15, 0.69; 0.20	0.25	−0.18, 0.68; 0.26
Sensitivity analysis^c^						
Only those who required a reminder: number of reminders = 0	0/61 (0)	1/80 (1)				
Number of reminders = 1	26/61 (43)	34/80 (43)				
Number of reminders = 2	10/61 (16)	16/80 (20)				
Number of reminders = 3	7/61 (11)	12/80 (15)				
Number of reminders = 4	7/61 (11)	8/80 (10)				
Number of reminders = 5	11/61 (18)	9/80 (11)	−0.22	−0.72, 0.28; 0.38	−0.15	−0.65, 0.36; 0.56
Secondary analysis^d^						
Time to complete questionnaire, median days	9.0	11.0	0.84	0.64, 1.10; 0.22	0.88	0.67, 1.15; 0.34
Sensitivity analysis^d^						
Only those who required a reminder: time to complete questionnaire, median days	22.5	19.0	1.12	0.78, 1.60; 0.54	1.08	0.75, 1.55; 0.67

Data are reported as number/total (percent), unless otherwise indicated. All primary analyses are intention-to-treat analyses. Sensitivity analyses included only those participants who required a reminder (excluded those who responded before a reminder was due and those who opted to complete the Patient Health Questionnaire (PHQ-9) over the phone before being sent any form of questionnaire) and, in addition to this, removing two outliers in terms of length of time it took to return the questionnaire (>80 days)

^a^Analyses adjusted by participant sex, baseline age and Patient Health Questionnaire (PHQ-9) score, main trial allocation arm, questionnaire method sent (paper or online)

^b^Logistic regression analysis of pre-call group allocation on whether the 8-month questionnaire was completed or not (odds ratio)

^c^Linear regression of pre-call group allocation on total number of reminders (linear regression coefficient, *b*). In the sensitivity analysis that includes only those who required an email reminder (i.e., excluded those who responded before a reminder was due and those who opted to complete the Patient Health Questionnaire (PHQ-9) over the phone before being sent a questionnaire), there was one participant who required an email reminder, but it was not sent due to administrative error

^d^Cox regression of pre-call group allocation on time to complete the questionnaire in days since the questionnaire was sent to the participant (hazard ratio). Zero values are not permitted in the time-to-event analysis, and so 0.5 days were allocated for participants who completed the questionnaire the day it was sent to them, while 91 days were allocated to the 18 participants that did not complete the questionnaire. For median: control *n* = 109 (sensitivity *n* = 54); urgent email *n* = 104 (sensitivity *n* = 69)

## Discussion

Participant retention is a methodological concern in trials, and so identifying successful strategies to improve follow-up questionnaire completion is important. This may be especially pressing in trials at greater risk of attrition, such as those with longer follow-up periods [3, 4], those including telehealth interventions [5], and those involving participants with depression [6, 7]. In three response rate intervention studies embedded within a 12-month telehealth trial for participants with depression (The Healthlines Study), there was no indication that any of these interventions improved overall response rate. However, there was evidence that pre-calling participants had some beneficial effects. Compared with those receiving no advance notification, pre-called participants at eight months post-randomization were less likely to require a questionnaire reminder, required fewer reminders, and returned the follow-up questionnaire about eight days earlier than controls. This strategy might be helpful when the timing of outcome completion is important.

Contrary to expectation, none of the response rate interventions appeared to boost the overall number of completed questionnaires, although this is probably because of very high response rates, creating ceiling effects. The most likely explanation for the unexpectedly high and sustained response rates amongst this group of participants is that the study team were already doing many of the things that, according to systematic reviews [9, 10], tend to result in better response rates. This use of multiple strategies to ensure participant retention reflects recommendations in existing literature [22, 23], particularly with participant groups that are difficult to recruit and retain [24]. In these ways, we had already optimized retention and response to some degree prior to introducing the response rate interventions. Nonetheless, we devised and carried out these studies because of an early indication at the start of the 8-month follow-up that response rates were below those of the previous follow-up and below the trial protocol target. Since participants were recruited from 43 practices over the course of approximately one year, there was overlap between the 8- and 12-month follow-ups; the response rate to the 8-month follow-up could not be determined prior to the start of the 12-month follow-up. Moreover, the pre-calling intervention from Study 1 was adopted as a standard procedure for all participants at the 12-month follow-up. This latter strategy, coupled with the multiple other response-boosting tactics already employed in the host trial, might have accounted for the better than expected response rate at 12 months.

Other studies have also failed to achieve an improved response rate, but did similarly enhance completion rates. In a recent study that included pre-notification calls as an embedded intervention within the host trial, there was a small, but non-significant effect on response rates [25]. Interestingly, however, the pre-called group had a higher response rate at the next scheduled follow-up, suggesting a carry-over effect from the previous telephone contact. It is possible that this occurred in the current results of Study 2, since the same group of Bristol-based participants who received a pre-call at the 8-month follow-up were involved in the team photo study at 12 months. This might have diluted any potential effect of the photograph intervention. Indeed, 48 % of pre-called participants required a reminder and took about 14 days to return the questionnaire in Study 1 (versus 62 % and 22 days, respectively, for control participants), which closely aligns with the figures for both the intervention (49 % and 14 days) and control (46 % and 13 days) groups in Study 2. Furthermore, another study demonstrated a similar carry-over effect for those who received a questionnaire with a colour photograph compared with those who received the black and white version in two waves of subsequent questionnaires [14]. Since the photograph intervention occurred at the final follow-up in the Healthlines host trial, it is not possible to examine whether this was also the case in our study. It is, nonetheless, plausible that these latter less resource-intensive strategies in isolation could be as effective at bringing about similar responding benefits as the more effortful pre-calling tactic. In a second example, Ashby and colleagues [26] reported a non-significant increase in response rates of participants who were sent an email or text message (or both) just after being posted their next questionnaire, but did observe a faster response time. Completion rates in this embedded trial (89 % overall) approached ceiling levels, just like our follow-up time points. Perhaps the previously documented benefit on completion rates of pre-notification, as well as the inclusion of a photograph or email subject line intervention [9, 10], applies to studies with poorer response rates to begin with.

As we noted earlier, different response rate strategies might bring comparatively different benefits and costs, in which a trade-off exists between the effectiveness of response rate strategies and the resources required to implement these tactics. In line with previous reviews [9–12], the results of the current studies suggest that the resource-intensive strategy – pre-calling participants – was more effective than the more easily implemented distinctiveness-enhancing strategies (including a personalized photograph and using an urgent action email subject line). However, there are two related, but opposing, issues to consider. First, despite the reduction in reminders and faster completion time, pre-calling participants probably did not result in an overall net benefit, since additional researcher time was required to contact these participants by telephone prior to sending out the questionnaire. Although we did not actually measure these additional researcher costs, the majority of the participants in this study required multiple telephone attempts to establish contact, sometimes outside of normal working hours. It is likely, therefore, that the effort expended at the outset to pre-call participants offset the reduction in questionnaire reminders. It would be worthwhile for future studies to quantify such cost-benefit trade-offs. Second, given the elaborate reminder protocol adopted in the main trial, the researchers perceived a substantial benefit from the reduction in the number of questionnaire reminders required. Not only did the reminder protocol result in a heavy administrative workload, but researchers also disliked the feeling of chasing and nagging participants to complete questionnaires; a feeling that they perceived to be shared by the participants themselves. Therefore, while it was laborious to successfully complete the pre-notification telephone call, the researchers felt that the benefits of this outweighed the later reduction in questionnaire reminders.

### Strengths and limitations

There are two key advantages shared by these three studies. Firstly, we employed an embedded study design, in which the response rate interventions occurred within an ongoing, complex, pragmatic trial (The Healthlines Study). Bower and colleagues [27] note that embedded studies are ‘the most robust test of the effectiveness of a recruitment or retention method’, since they permit less biased and more externally valid evaluations of such strategies, but acknowledge that these studies are quite rare. A second shared advantage is that the current studies include a joint assessment of responses to both postal and electronic questionnaire completion. Thus, while it remains an open question, whether the results of some previous response rate intervention effects were replicable across different questionnaire completion methods, our results hold across paper-based and online surveys.

A number of limitations of these studies should be taken into consideration. Firstly, owing to the practical constraints of the embedded study designs, we did not calculate power calculations *a priori* for these three studies. It is, therefore, possible that the studies did not include an adequate sample size to detect differences in effects. This is especially the case in Study 3, in which only 61 % (141/231) of randomized participants required a study reminder. Yet, both intention-to-treat and sensitivity analyses, whereby only participants exposed to the treatment allocation – a condition that is independent of group allocation, and so unlikely to introduce bias – revealed the same pattern of findings. In a similar embedded study, the authors calculated *post facto* that 4,000 participants would be required to detect a small but significant response rate effect – a figure that they state would be difficult for studies to achieve [27]. However, as these authors recommended, publishing such embedded studies will enable future meta-analyses. Secondly, our results might not be generalizable to other kinds of trial or patient population. Unlike the majority of previous trials, participants in the current studies were given the option of completing either postal or online questionnaires, according to their preference. This might have contributed to the high response rates we observed. In addition, participants who volunteer to take part in a telehealth trial might differ from other patient populations in terms of their accessibility to and confidence using technologies [28]. Finally, the pre-calling study used alternate allocation of participants to the two study arms, which was, therefore, not truly random. While this is a methodological drawback, Table 1 clearly shows that the two groups were very well balanced across all characteristics, and comparably so with Studies 2 and 3, which did make use of simple randomization.

## Conclusions

Embedded response rate studies within a host trial are a rigorous method of evaluating strategies for improving participant retention and responding, but remain relatively rare. Three studies embedded in a telehealth randomized controlled trial for depression revealed no benefit to overall response rates, regardless of employing a resource-intensive strategy (pre-calling) or more easily implemented distinctiveness-enhancing strategies (including a team photograph or an action-oriented email subject line). In many instances, however, it is important to collect primary outcome data in a timely manner, so that treatment effects can be confidently attributed to the intervention or because of tight deadlines around study closure. Delayed responding also increases costs incurred in sending out reminders, as well as administrative time and effort. The current results suggest that pre-calling participants, but not including a research team photograph or action-oriented email subject lines, might be a successful strategy for realizing these benefits.

## Additional files

Additional file 1:
**Examples of response rate interventions from Studies 1–3.** (DOCX 242 kb) Additional file 2:
**CONSORT checklist for Studies 2 and 3.** (DOC 224 kb)

## Abbreviations

CI: confidence interval
CONSORT: Consolidated Standards of Reporting Trials
HR: hazard ratio
OR: odds ratio
PHQ-9: Patient Health Questionnaire
