# Supplementary material for: Increasing follow-up questionnaire response rates in a randomized controlled trial of telehealth for depression: three embedded controlled studies
Source: Trials. 2016 Feb 24;17:107. doi: 10.1186/s13063-016-1234-3 (PMC4765058; doi:10.1186/s13063-016-1234-3)
Supplement: Additional file 1: — Examples of response rate interventions from Studies 1–3. (DOCX 242 kb) [file 13063_2016_1234_MOESM1_ESM.docx]

## Additional file 1

### Study 1: Pre-calling script excerpt

“Hi <Forename>,

This is <Researcher Name> calling from The Healthlines Study at the University of Bristol. This is just a quick courtesy call to let you know that the next questionnaire is due for the study. I see that you completed the last questionnaire online <by post> and I just wanted to check if this is still the easiest way for you to complete this next questionnaire.

<After confirming best/preferred completion method:>

Ok, great. We’d be really grateful if you could try to get the questionnaire back to us in the next 10 days. Do you think that would be possible?

Just before I go, could I double check that we still have the correct address and email for you?

<After confirming address and email:>

Ok, <Forename>, thanks again for participating in this research. We really appreciate you taking the time to complete our questionnaires and look forward to getting your answers back in the next week or so.”

### Study 2: Cover letter with research team photo (postal questionnaire version)


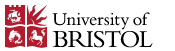

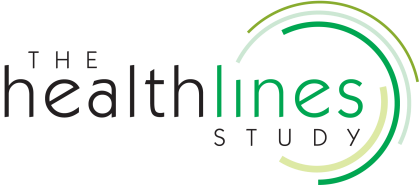


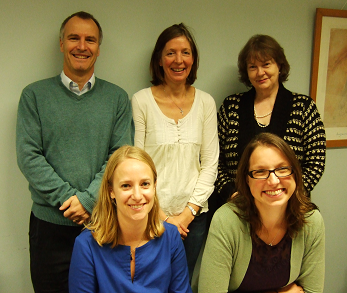


<Title> <Forename> <Surname>

<Address1>

<Address2>

<Address3>

<Postcode>

The Healthlines Study Research Team

Back row: Chris Salisbury, Katy Garner, Frederika Collihole

Front row: Louisa Edwards, Clare Thomas

<Date>

Dear <Forename>,

**The Healthlines Study: Researching Different Ways of Providing Support for Health and Well-being**

Once again, we’d like to thank you for your continued participation in The Healthlines Study. It is now time to complete the **final questionnaire** for the study! This is the last questionnaire we will ask you to complete for the study, and marks the end of your participation in The Healthlines Study. Once we receive your completed questionnaire, we will email you a ‘thank you’ note that will also include some additional information about what happens next.

Since you told us you would like to complete the other questionnaires by post, we decided to send you this final questionnaire by post again. However, if you would prefer to complete this questionnaire using our secure study website, please do let us know.

Enclosed is the study questionnaire, which will take you about 30-40 minutes to complete. This includes many of the same questions from the second study questionnaire, but we would still like you to try to answer all of the questions. The answers you give and the information you provide are both important and relevant. Even if you haven’t made many changes to your lifestyle, the information you provide is extremely helpful for us.

*Please remember that all questions are completely confidential. We will not share your answers with your doctor or other members of staff at your surgery or health centre. The only exception is if we have concerns about your safety, in which case we will tell your doctor. However, we will always try and contact you first to check it is ok for us to do this. We want to reassure you that your answers will not affect the care you receive from your doctor or nurse(s).*

Please complete this questionnaire and return it in the envelope provided (no stamp required) as soon as possible. **We would be most grateful if you could return this to us within the next 10 days or so.**

If you require any further information, please do not hesitate to contact us (Telephone: <Number>; Email: <Email>@bristol.ac.uk). Once again, thank you for being involved in the study.

With best wishes,

<Researcher’s signature>

<Researcher Name>

The Healthlines Study

University of Bristol

School of Social and Community Medicine Canynge Hall

39 Whatley Road

Bristol

BS8 2PS

### Study 3: Email reminder excerpt

Subject line: The Healthlines Study – ACTION REQUIRED

Dear <Forename>,

Thank you for your continued participation in The Healthlines Study. We emailed you the link to the **final study questionnaire** about a week ago. **We would be very grateful if you could please complete this in the next few days.** If you would prefer to complete this final questionnaire by post, however, please do let us know.

This is the last questionnaire we will ask you to complete for the study, and marks the end of your participation in The Healthlines Study. Once we receive your completed questionnaire, we will email you a ‘thank you’ note that will also include some additional information that you might find of interest.

I've copied the original email we sent you into the space below about how to gain access to and complete the online questionnaire:

As before, the online questionnaire should take about 30-40 minutes to complete. This questionnaire includes many of the same questions from the third study questionnaire, but we would still like you to try to answer all of the questions. The answers you give and the information you provide are both important and relevant. Even if you haven't made many changes to your lifestyle, the information you provide is extremely helpful for us.

*Please remember that all questions are completely confidential. We will not share your answers with your doctor or other members of staff at your surgery or health centre. We want to reassure you that your answers will not affect the care you receive from your doctor or nurse(s).*

<…>

Once again, many thanks for being involved in The Healthlines Study.

Sincerely,

<Researcher’s name>

The Healthlines Study
